# Inclusivity is child’s play: pilot study on usability, acceptability and user experience of a sensory-motor PC game for children with cerebral palsy (GiocAbile)

**DOI:** 10.1186/s13052-024-01830-7

**Published:** 2024-12-20

**Authors:** Alessandra Consales, Emilia Biffi, Roberta Nossa, Simone Pittaccio, Fabio Lazzari, Matteo Malosio, Matteo Lavit Nicora, Giovanni Tauro, Davide Felice Redaelli, Atul Chaudhary, Eleonora Diella, Matteo Valoriani, Francesca Fedeli, Odoardo Picciolini, Maria Lorella Giannì, Matteo Porro

**Affiliations:** 1 Department of Clinical Sciences and Community Health, Dipartimento di Eccellenza 2023-2027, University of Milan, Milan, Italy; 2 Scientific Institute, IRCCS Eugenio Medea, Bosisio Parini, Italy; 3 Institute of Condensed Matter Physics and Technologies for Energy (CNR-ICMATE), National Research Council of Italy, Lecco, Italy; 4 Institute of Intelligent Industrial Technologies and Systems for Advanced Manufacturing (CNR-STIIMA), National Research Council of Italy, Lecco, Italy; 5 Industrial Engineering Department, University of Bologna, Bologna, Italy; 6 Fifthingenium, Milan, Italy; 7 FightTheStroke Foundation, Milan, Italy; 8 Pediatric Physical Medicine & Rehabilitation Service, Fondazione IRCCS Ca’ Granda Ospedale Maggiore Policlinico, Milan, Italy; 9 NICU, Fondazione IRCCS Ca’ Granda Ospedale Maggiore Policlinico, Milan, Italy

**Keywords:** Accessibility, Cerebral palsy, Design4All, Disability, Gamification, Neuromotor impairment, Videogames

## Abstract

**Background:**

The use of video games in rehabilitation settings is gaining increasing popularity. However, the lack of commercial video games suitable for children with disabilities and the disappointing user experience of serious games limit their applicability. The aim of this study was to assess the usability, acceptability and user experience of GiocAbile, an active video game for children with cerebral palsy (CP).

**Methods:**

This multicenter pilot observational study was conducted from May to September 2022 at the participating institutions, and enrolled school-aged children affected by CP. Enrolled children played GiocAbile in single-player mode for one hour. The gaming experience was evaluated through self-assessment questionnaires. Non-parametric correlation analysis was conducted to examine the influence of motor and cognitive abilities (GMFCS, MACS, ICF) on declared usability and acceptability.

**Results:**

Nineteen children (9.01 ± 1.95 years, 63.1% male) with mild to severe CP were enrolled. The 100% of respondents expressed satisfaction and fulfillment associated with gameplay, with no reports of frustration or disappointment. The 83% would recommend the game to a friend. The controllers were generally deemed easy to use and maneuver, with very few reports of discomfort associated with their use. No correlations were found between usability/acceptability levels and measures of impairment (i.e., GMFCS, MACS, and ICF scales), while cognitive impairment positively correlated with satisfaction during gameplay.

**Conclusions:**

GiocAbile is an accessible, user-friendly and enjoyable tool for children with CP, regardless of level of impairment. Based on existing literature, we hypothesize that GiocAbile may improve motivation, participation, and rehabilitation outcomes in children with CP, although further studies are needed to confirm our hypothesis.

**Supplementary Information:**

The online version contains supplementary material available at 10.1186/s13052-024-01830-7.

## Background

Cerebral palsy (CP) encompasses a range of permanent, but not immutable, physical disabilities caused by a brain lesion occurring during a highly vulnerable phase of brain development (i.e., the first 2 years of life). CP affects thousands of children each year globally [1–3]. The associated movement and coordination impairments can significantly limit a child’s ability to walk, manipulate objects, and independently perform daily tasks. Similarly to their able-bodied peers [4], play is pivotal for children with CP, serving as a fundamental means of entertainment and socialization, thereby contributing to cognitive and social development. In recent years, technological advancements have expanded the range of games available for children, with video games gaining increasing popularity [5]. In alignment with contemporary trends, including video games in the rehabilitation plan for children with neuromotor disabilities such as CP may represent a promising avenue for therapeutic interventions [6].

Active video games, which incorporate physical movement into gameplay, have proven effective in promoting motor improvements and boosting motivation in children with neuromotor disabilities [7], and their use as a home-based intervention further emphasizes the practicality of integrating gaming technology into children’s daily routines [8].

While many studies [6, 8, 9] reported the effectiveness of commercially available video games in virtual rehabilitation, it should be noted that such games are primarily developed for able-bodied individuals, not considering the specific needs of people with disabilities. Adaptations are therefore necessary, particularly in terms of game controllers, and the lack of flexibility for customization by therapists may pose significant limitations to their therapeutic use. Various “hybrid” solutions [10–12] (i.e., commercial hardware, rehabilitation-specific software) and rehabilitation-specific software and hardware [13, 14] have been proposed and tested, yet video games created for medical purposes are typically repetitive and lack engaging storylines and characters, often resulting in decreased patients’ motivation in rehabilitation [10, 14].

In response to these considerations, there is a pressing need to find a balance between developing customized video games accessible to children with neuromotor disabilities and making them captivating enough to stimulate interest and engagement. To this end, our previous research [15] assessed the interests of children with neuromotor disabilities regarding video games and identified the requirements for an accessible and inclusive gaming experience (i.e., intuitive controllers with resemblance to daily objects that allow bi-manual skill training and functional movement training; compatibility with computers and consoles; possibility of cooperative gameplay; action/adventure games possibly organized in minigames with characters easy to identify with; progressive levels; bright primary colors; short gaming sessions of 20–30 min; affordable pricing).

Primary aim of the present study was to pilot test GiocAbile, a novel active video game designed to be accessible for children with neuromotor disabilities (e.g., CP) thanks to controllers designed according to the Design4All principles [16]. The present study aimed to assess GiocAbile’s usability, acceptability and user experience among school-aged children with CP to provide insights into its practicality and overall satisfaction.

## Materials and methods

### Study design and setting

The present study was a multicenter, observational, cross-sectional pilot study conducted from May to September 2022. The study was coordinated by the Pediatric Physical Medicine and Rehabilitation Service, Fondazione IRCCS Ca’ Granda Ospedale Maggiore Policlinico, Milan, Italy in collaboration with IRCCS E. Medea, Bosisio Parini, Italy. Partners FifthIngenium and the National Research Council of Italy handled product calibration and technically supervised the video game application. The study was conducted in a hospital setting (i.e., the two research centers involved). The study was approved by the Ethics Committee of Milano Area B (No 817; date of approval: 20/07/2021) and by the IRCCS Medea Ethics Committee (Prot. N. 89/21-CE; date of approval: 12/11/2021). Participation in the present study was proposed to parents/legal guardians during routine follow-up visits. Written informed consent was provided by parents/legal guardians of the children enrolled.

### Study population and initial evaluations

We enrolled school-aged children with CP, among those regularly followed at the participating centers.

The initial assessment aimed to evaluate the participants’ overall functioning and their ability to engage in play activities. Trained examiners from the two centers conducted the evaluation using the International Classification of Functioning, Disability, and Health (ICF) framework. This comprehensive assessment considered not only body structures and functions, but also levels of activity and participation, focusing on Chaps. 1 (mental and cognitive functions) and 7 (musculoskeletal and motor functions) of the Body Functions domain of the ICF.

Regarding the motor (Chap. 7 of the ICF) and cognitive (Chap. 1 of the ICF) functioning, children classified as 0 (no impairment), 1 (mild impairment), 2 (moderate impairment) and 3 (severe impairment) were included, while those classified as 4 (complete impairment) were excluded due to severe motor, gestural and cognitive impairment hindering task execution and understanding.

The initial evaluation also included:

- Gross Motor Function Classification System (GMFCS) [17]: a widely employed framework to assess the gross motor function of children and youth with CP. It comprises five levels (I to V), each denoting a distinct level of motor impairment, progressing in severity.
- Manual Ability Classification System (MACS) [18]: a system designed to classify the manual abilities of children with CP. It consists of five levels, ranging from Level I (limited limitations) to Level V (no effective hand use).

Finally, parents or legal guardians were asked to answer the Assistance to Participate Scale (APS) [19] questionnaire to assess the child’s autonomy and participation levels in play and leisure activities both at home and in social contexts. Questions were scored using a five-point Likert scale (1 = Unable to participate; 2 = Participates with my assistance at all stages of the activity; 3 = Participates after I have set him/her up and help at times during the activity; 4 = Participates with my supervision only; 5 = Participates independently). The APS total score, calculated according to Bourke-Taylor et al. [19], ranges from 0 to 100, where higher scores indicate less assistance required for the child to participate in the activity.

### Instrument

GiocAbile is a sensory-motor PC game specifically tailored for children with neuromotor disabilities like CP. The name “GiocAbile” is a blend of the Italian words “gioco” (play) and “abile” (able). The combination of these terms conveys the idea of a video game that is both enjoyable and accessible, particularly for individuals with CP. Indeed, GiocAbile addresses the need for both rehabilitation and integration of children with CP, offering an enabling and inclusive gaming experience. GiocAbile can be played while sitting on a chair, in a wheelchair or standing, depending on preference and ability. The GiocAbile system comprises both software (the video game) and hardware components (PhiCube and PlayCuff).

### Video game

**Fig. 1:**
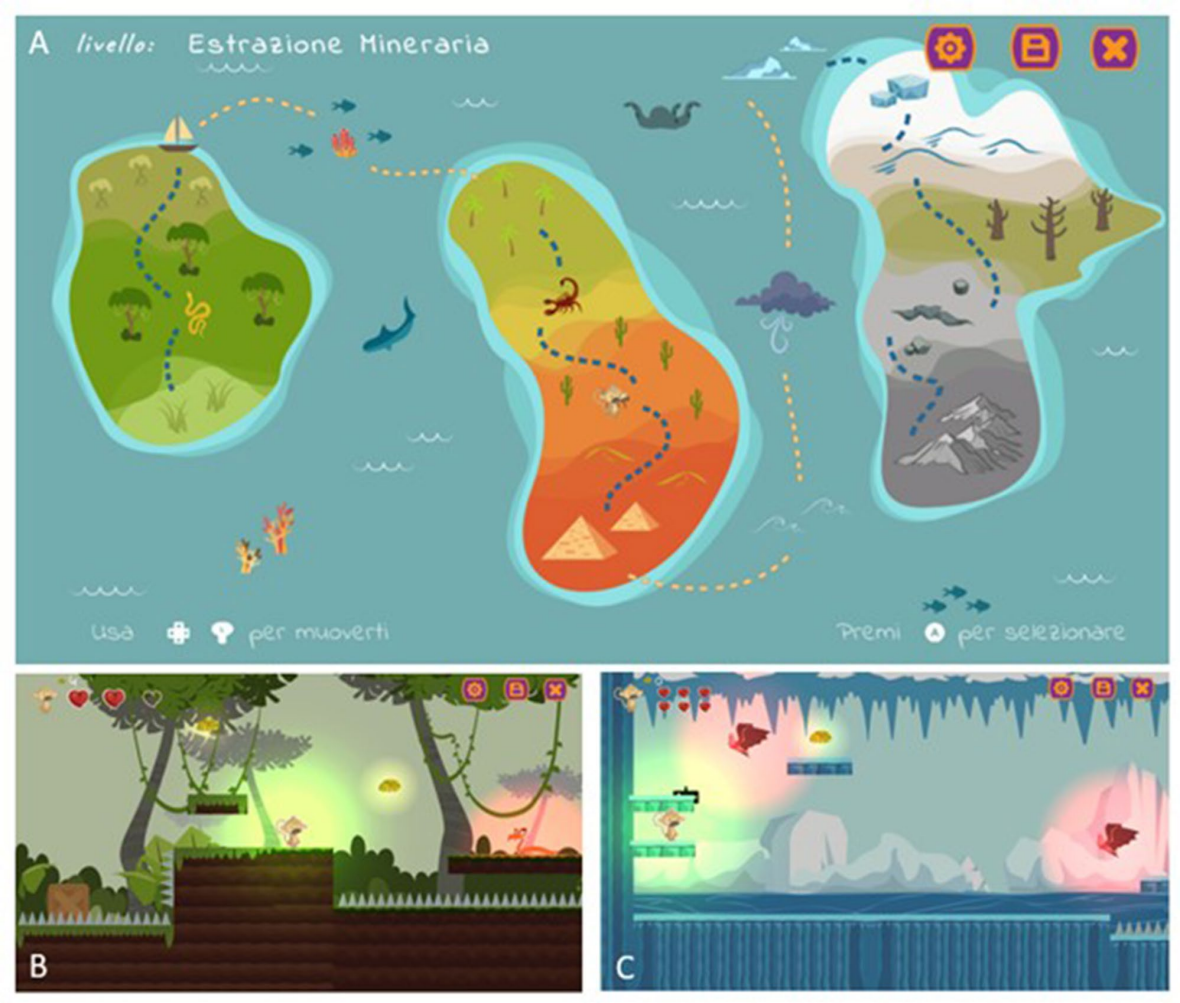
The video game: (**A**) island structure of the video game (**B**) Jungle platform-game environment and (**C**) Lands of ice platform-game environment

**Fig. 2:**
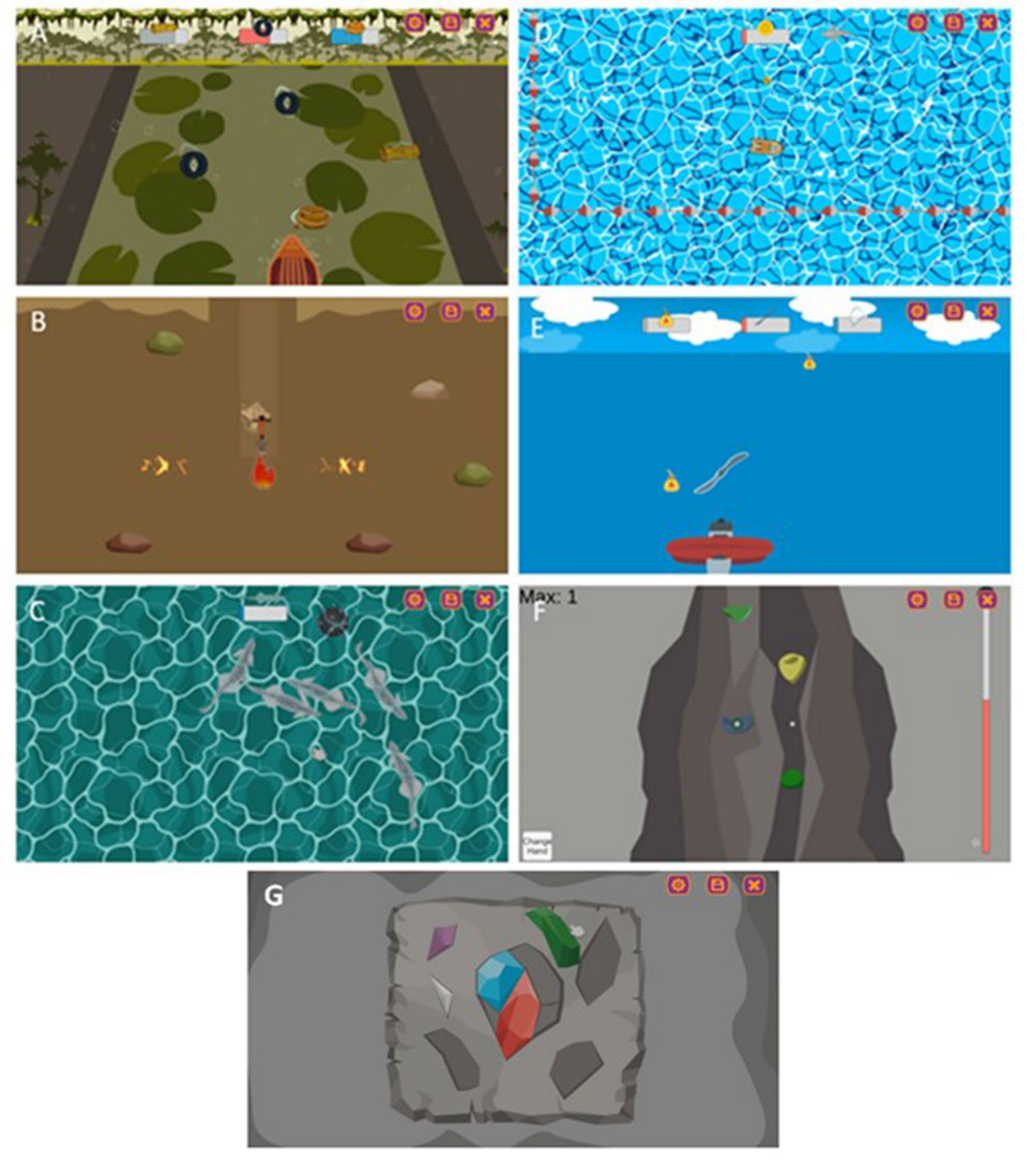
Minigames: (**A**) Canoeing; (**B**) Drill; (**C**) Fishing; (**D**) Boat; (**E**) Flight; (**F**) Climbing; (**G**) Crystal assembly

The software features a collection of game activities stimulating various motor and cognitive abilities, with the possibility of a multiplayer mode focused on cooperative, non-competitive, gameplay. The video game features an island structure (Fig. 1A) consisting of a series of platform games where the player must navigate through different levels of increasing difficulty, each set in unique environments, such as the Jungle or Lands of ice (Fig. 1B and C). These platform games are interspersed with minigames grouped according to specific body functions, which vary in difficulty levels, ranging from simple segmental movements to more complex articulated motions (Fig. 2). The final challenge is the assembly of a crystal, where the participant must piece together crystal fragments like a puzzle (Fig. 2G). The main character of the video game is Mako the monkey (Figs. 1 and 2).

### PhiCube

**Fig. 3:**
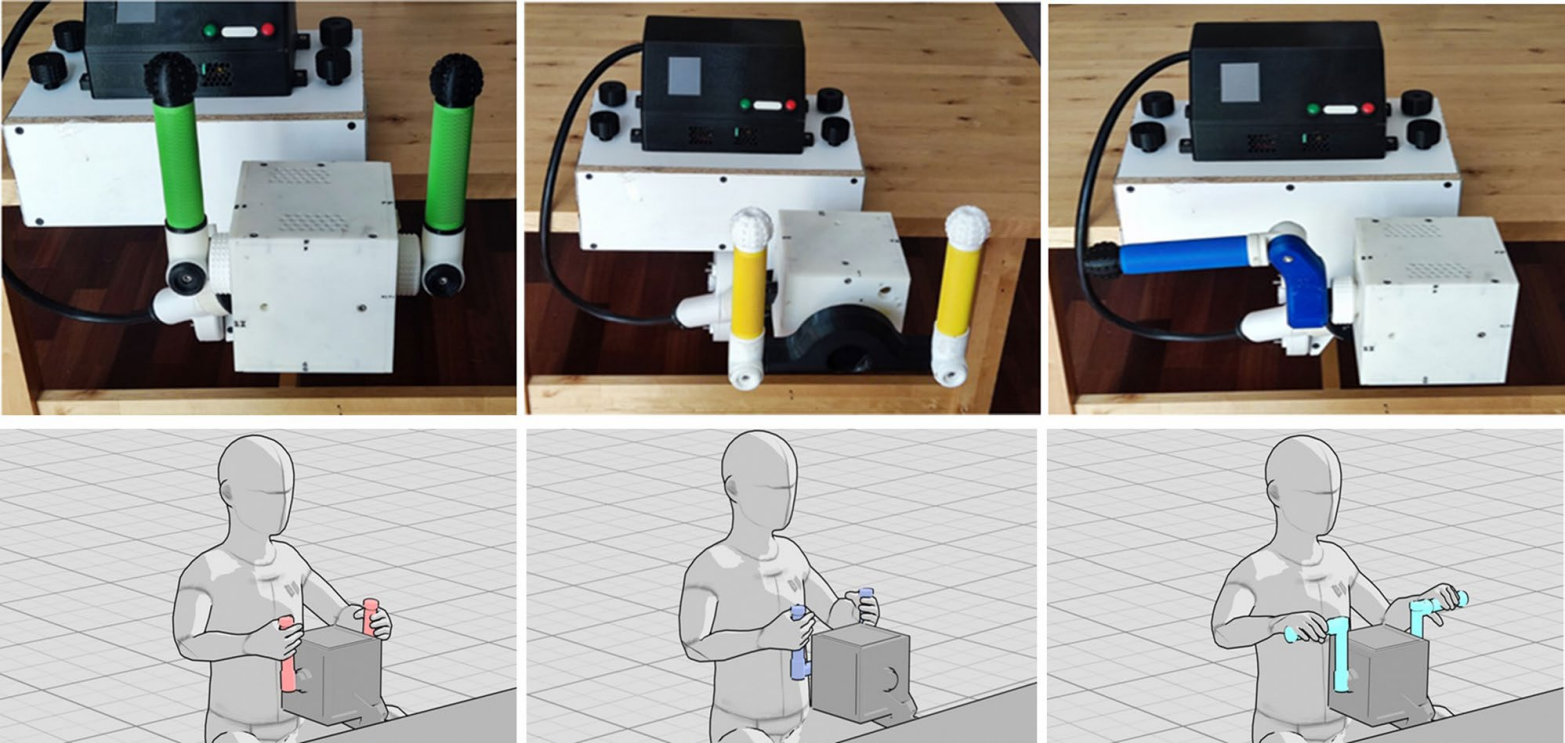
PhiCube with the manipulanda: levers (left), steering wheel (center), cranks (right)

PhiCube (Fig. 3) is a modular and portable robotic bilateral haptic interface, characterized by high flexibility of use [20]. It allows children to perform different bilateral motor gestures required for different game scenarios.

PhiCube consists of a motorized unit, a control unit and a set of manipulanda (i.e., steering wheel, levers, cranks) (Supplementary Figs. 1 and 2). The core of PhiCube is a robotized central unit equipped with two motorized rotary axes. The axes are equipped with appropriate mechanical interfaces, placed on the two opposite sides of the central body, and with a quick connection for different manipulanda. The Control Unit allows to select the manipulanda and the corresponding control strategies. Manipulanda are made of plastic by additive manufacturing and have handle grips certified for use on bicycle handlebars. With PhiCube, the user can interact with the game in a bilateral way and, thanks to the chosen set of manipulanda, can execute various motor gestures, involving shoulder, elbow and wrist joints in different ways.

### PlayCuff

**Fig. 4:**
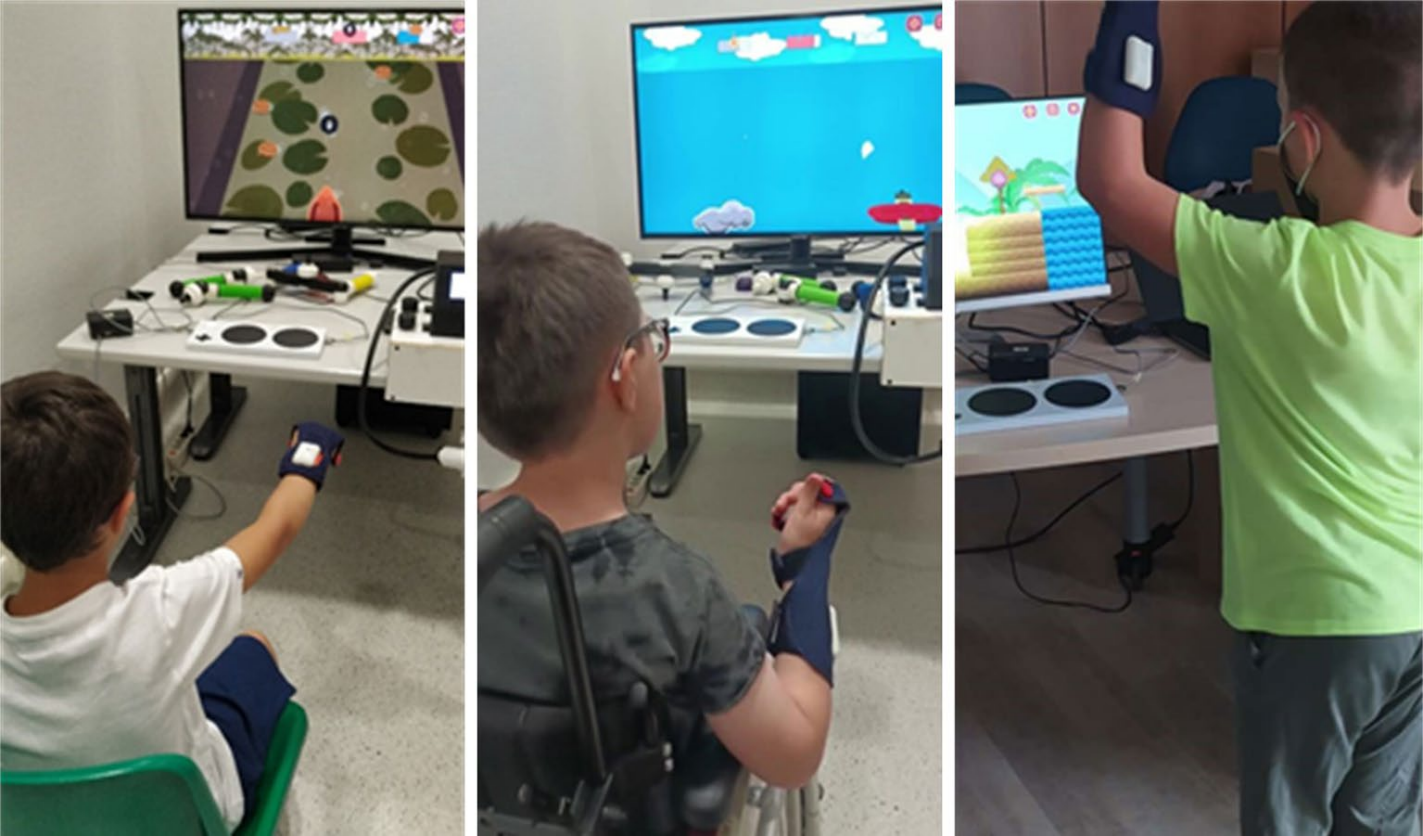
Children playing GiocAbile using PlayCuff

PlayCuff is a wireless sensorized orthosis that can be worn like a fingerless glove (Suppl. Figure 3). It consists of a double layer of soft neoprene fabric and a separate hand piece. It can be worn by both right-handed and left-handed people and is available in three different sizes to fit children of different ages. PlayCuff implements an orthotic function thanks to two NiTi pseudoelastic alloy elements sewn in between the fabric layers to control wrist posture. The characteristics of this material allow proprioceptive feedback and dynamic joint extension, thereby improving motor control, especially in children with dyskinetic traits [21, 22]. PlayCuff is also equipped with two battery-powered inertial sensors (IMU) and a microcontroller that classifies forearm and wrist gestures in real-time, achieving a frequency of 20 Hz, with very high accuracy (94% and 99.5%, respectively). The recognizable movements include waving the limb up, down, right and left, pronating and supinating the forearm, extending and flexing the wrist or deviating it laterally. Additionally, static positions like pointing upwards, horizontally or downwards can be classified. Based on such gesture recognition capacity, the device can be used to play video games (Fig. 4), controlling the avatar’s actions and making choices within the game menus.

### Testing session and outcome measures

During the testing phase, participants had the opportunity to experience the video game in single-player mode for a total test duration of up to 1 h, as suggested by the American Academy of Pediatrics [23]. Short breaks (one minute) were scheduled every 20 min as advised by the American Optometric Association [24] and the American Academy of Ophthalmology [25].

Children engaged with GiocAbile for approximately 30 min using PhiCube and another 30 min using PlayCuff. Following their gameplay session, children’s gaming experience was assessed through the System Usability Scale (SUS) [26], the Technology Acceptance Model 3 (TAM3) [27] and an ad hoc questionnaire developed by the authors. This phase lasted from 30 to 60 min.

The SUS, a five-point Likert scale questionnaire, evaluates system usability, with a total score ranging from 0 to 100.

The TAM3 evaluates the acceptability of the system according to 14 constructs. Considering the type of device and the assessors (i.e., children) we reduced the assessed dimensions to 8 (i.e., perceived usefulness, perceived ease of use, self-efficacy, perception of external control, playfulness, anxiety, enjoyment and behavioral intention), each rated on a scale of 1 (totally disagree) to 7 (totally agree) [27].

The ad hoc questionnaire (Suppl. Table 1), specifically designed to assess GiocAbile’s software and hardware features, used a five-point Likert scale to evaluate children’s appreciation of the story, character, settings, and sounds proposed.

Language revisions were applied to all questionnaires to enhance understanding for children. Additionally, graphical representations with emoticons were included alongside the Likert scales for improved clarity.

### Statistical analysis

Median and interquartile values (i.e., the difference between the 75th percentile and the 25th percentile) were calculated for GMFCS, MACS, ICF, APS and questionnaire scores. SUS total score was computed in Matlab 2022 according to Brooke et al. [26]. TAM3 mean score and constructs were computed in Matlab 2022 according to Venkatesh et al. [27]. For the ad hoc questionnaire, answers referring to the evaluation of PhiCube manipulanda (i.e., steering wheel, single and double crank, single and double lever) were aggregated. The questions regarding PlayCuff and PhiCube were divided in 5 constructs as follows: *I managed to use the controllers* (Usability); *I managed to get the controllers to do what I wanted* (Control); *Using the controllers was fun* (Enjoyment); *Using the controllers was disappointing* (Disappointment); *Using the controllers was complex* (Complexity).

A non-parametric correlation analysis was conducted to examine the relationships between GMFCS, MACS, ICF scores, and questionnaire results, aiming to identify influences of children’s motor and cognitive abilities on the declared usability and acceptability of GiocAbile.

Statistical analysis was performed using SPSS (Statistical Package for the Social Sciences, IBM SPSS Statistics for Windows, Version 21.0. Armonk, NY: IBM Corp.).

For all tests, a p value < 0.05 was considered statistically significant.

## Results

The study population included 19 children (mean age 9.01 ± 1.95 years, 63.1% males). Table 1 shows the results of the baseline evaluations. Median (interquartile value) APS total score was 66.5 (12), suggesting a moderate level of independence of the participants during play. Baseline evaluations and APS total score for single participant are reported in Supplementary Table 2.

**Table 1:** baseline evaluations of the study population

	%
**GMFCS**	
I	47.4
II	26.3
III	5.3
IV	15.8
V	5.3
**MACS**	
I	27.8
II	33.3
III	38.9
**Cognitive ICF**	
0	15.8
1	36.8
2	31.6
3	15.8
**Motor ICF**	
0	22.2
1	27.8
2	22.2
3	27.8

**Fig. 5:**
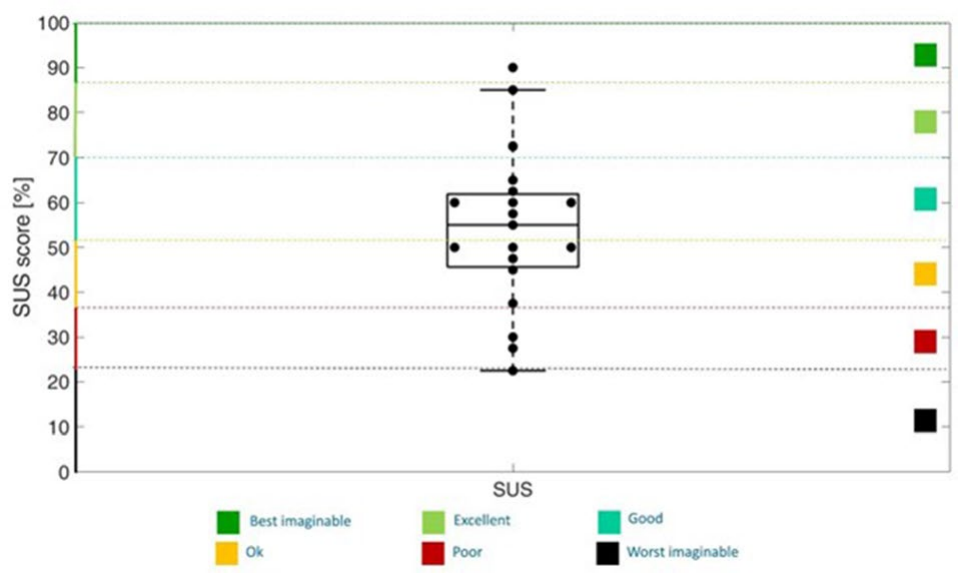
SUS results. SUS score is divided into “best imaginable” (dark green), “excellent” (light green), “good” (aquamarine), “ok” (orange), “poor” (red), and “worst imaginable” (black) according to Bangor, Kortum and Miller, 2009 [28]

The median (interquartile value) SUS score was 55 (15), indicating that children rated the system usability between “OK” and “Good"^24^. Figure 5 shows the SUS score distribution for each player.

**Fig. 6:**
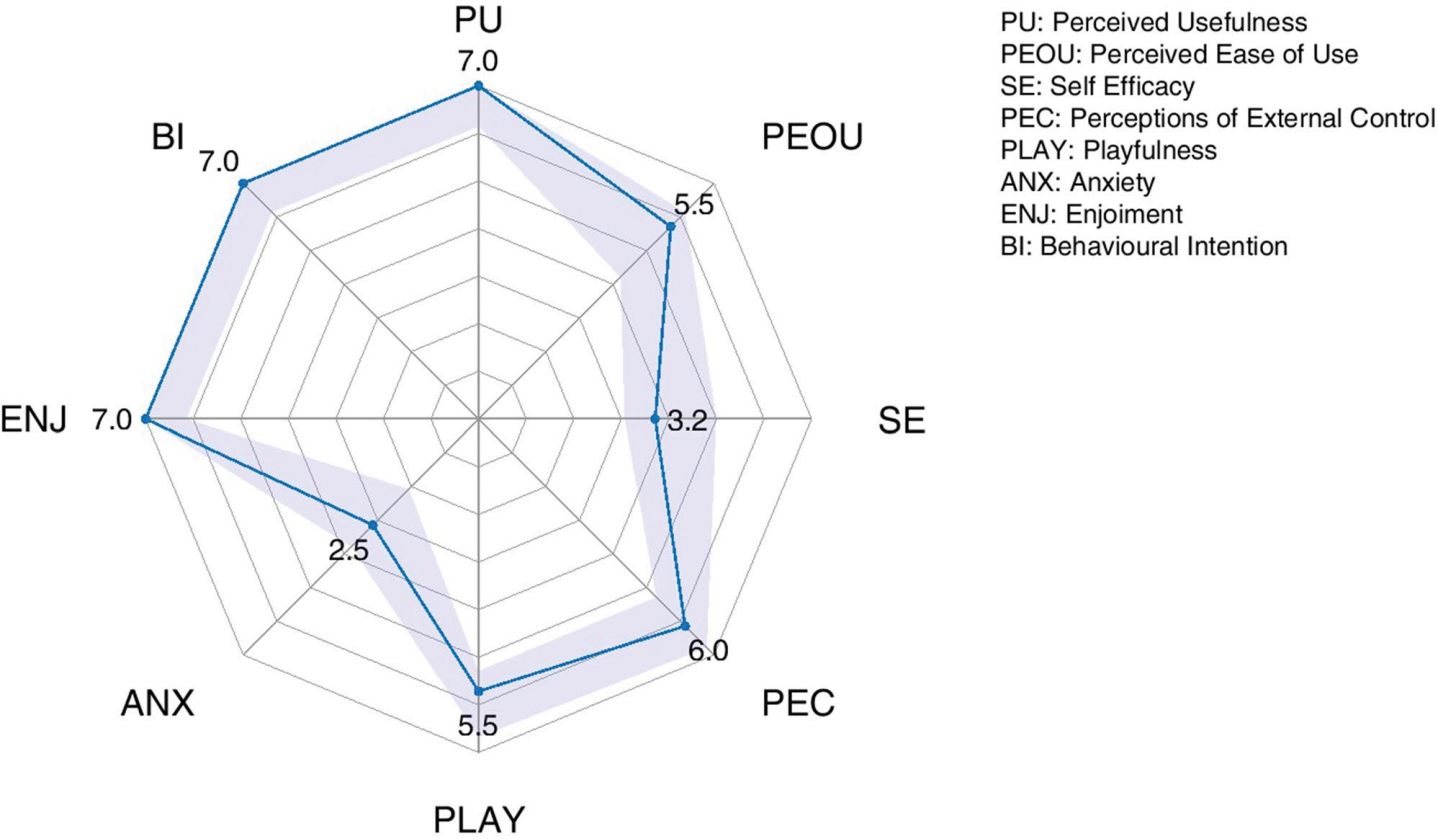
Constructs assessed by the TAM3. PU: Perceived Usefulness; PEOU: Perceived Ease of Use; SE: Self Efficacy; PEC: Perceptions of External Control; PLAY: Playfulness; ANX: Anxiety; ENJ: Enjoyment; BI: Behavioural Intention

Median (interquartile value) TAM3 score was 5.6 (0.54), indicating satisfactory acceptability. Figure 6 shows the dimensions assessed by the TAM3. Children found the system useful (PU), they enjoyed it (ENJ) and expressed the intention to use it again in the future (BI). They also found the system reasonably usable (PEOU), with positive perceptions of external control (PEC) and playfulness (PLAY), and without experiencing anxiety related to the system (ANX). However, the perception of self-efficacy (SE) was low, indicating a perceived lack of capacity to use the system.

**Fig. 7:**
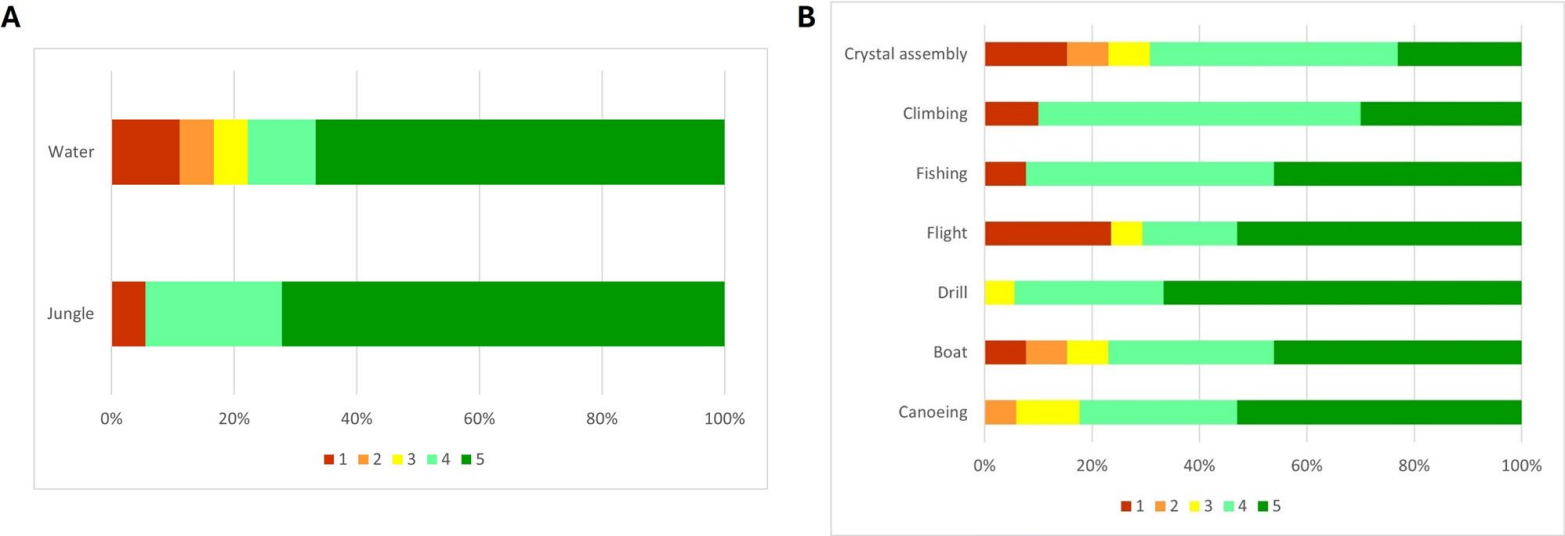
Relative percentages of answers to (**A**) “I liked the platform environments” and (**B**) “I liked the minigames”. 1-Totally disagree; 2-Disagree; 3-Neutral; 4-Agree; 5-Totally agree

According to the ad hoc questionnaire, participants were generally interested in the game storyline, with a median score (interquartile value) of 5 (1). They exhibited moderate identification with the character of the video game, Mako the monkey, with a median score of 3.5 (3). Nonetheless, they expressed a strong liking for it, with a median score of 5 (1), and they stated that they liked the sound during the games (median score 4 (1)). Children indicated overall appreciation for the platform-game environments, with a slight, albeit non statistically significant, preference for the Jungle environment (Fig. 7A). Among the minigames, Canoeing and Drill were the most frequently enjoyed, while Crystal assembly and Flight were less favored, although such differences did not reach statistical significance (Fig. 7B).

The findings from the ad hoc questionnaire (Supplementary Table 1) indicate a high level of satisfaction and positive reception towards PhiCube and PlayCuff. Regarding PhiCube, the “double lever” and the “steering wheel” emerged as the most enjoyable controllers, with nearly 80% of respondents expressing complete agreement with the statement “using the controllers was fun”. Overall, PhiCube manipulanda were deemed accessible, and the majority of children reported success in maneuvering them to achieve their desired actions. Regarding PlayCuff, 83% of participants found it helpful in reducing errors during gameplay and facilitating game completion. A significant proportion of children (72%) did not report experiencing any notable discomfort while using PlayCuff. In terms of overall satisfaction during gameplay, 100% of the respondents expressed feelings of fulfillment and satisfaction. None of the participants reported experiencing frustration or disappointment. Additionally, the majority (83%) of respondents indicated that they would recommend the game to a friend.

### Correlation results

The median scores of SUS and TAM3 did not correlate with GMFCS, MACS, and ICF scores. Furthermore, GMFCS, MACS and ICF scores did not correlate with the controllers’ evaluation through the ad hoc questionnaire. However, MACS demonstrated a positive correlation with the *Perceived Ease of Use* dimension of the TAM3 (Spearman’s rho = 0.470; *p* = .05) and a negative correlation with the *Perceptions of External Control* dimension (Spearman’s rho=-0.577; *p* = .01). Cognitive ICF positively correlated with the statement *I felt satisfied while playing* of the ad hoc questionnaire (Spearman’s rho = 0.551; *p* = .04). Finally, the APS total score positively correlated with the *Enjoyment* construct of the ad hoc questionnaire referred to PhiCube (Spearman’s rho = 0.530; *p* = .029), while it correlated negatively with the *Usability* construct of the ad hoc questionnaire referred to PlayCuff (Spearman’s rho=-0.554 ; *p* = .017).

## Discussion

Our pilot study findings suggest that GiocAbile is an accessible, user-friendly and enjoyable video game for children with CP, irrespective of their level of impairment. To the best of our knowledge, no prior research has evaluated whether and how gaming experience is influenced by motor or cognitive impairment in children with CP.

The high level of satisfaction reported by participants in our study is in contrast with previous studies in the literature. For instance, in the study by Gerber et al. [14], who tested eight different exergames for upper limb training with the YouGrabber^®^ system, participants found the training boring due to a limited number of games, a perceived lack of variety, unimpressive design, and a shortage of challenging and competitive elements. Moreover, parents frequently reported their children to need extrinsic motivation to continue the training. Likewise, experimentations with the gaming software package OpenFeasyo [29], featuring four customizable minigames, failed to show increased motivation in rehabilitation following the implementation of the gaming intervention [10]. The authors speculated that the relatively simple design of the video game might have been at least partially to blame [10]. In our study, children showed appreciation for both the video game avatar (Mako the monkey) and the game environments. Additionally, a high percentage of participants expressed the intention to use the video game again in the future and stated that they would recommend it to a friend.

Contrary to most studies on serious games, studies evaluating the use of commercial video games in the rehabilitation of children with CP showed increased motivation [30] and preference for video games compared to conventional treatments [31]. A pilot study demonstrated that the video game Wii Sports Resort by Nintendo^®^ played using a slightly adapted Nintendo Wii controller could be used as a rehabilitation tool for children with CP, with participants showing improvements in gross motor function [9]. Likewise, Sandlund et al. tested the feasibility of EyeToy3 for Sony PlayStation2^®^ as a home-based intervention for children with CP and documented improvement in motor performance [8]. However, commercial video games are developed for able-bodied individuals, thus adaptations for children with disabilities are needed. Conversely, GiocAbile already features two game controllers specifically designed for children with CP: PhiCube and PlayCuff. Children participating in the present study found the controllers accessible, helpful and enjoyable. Interestingly, children who were more autonomous in play activities (as testified by higher APS scores) enjoyed PhiCube more but had more difficulties in using PlayCuff compared to children with lower APS scores. PhiCube’s manipulanda likely provided a more tangible and intuitive mode of interaction, aligning well with the autonomy levels of more independent children, while possibly being less entertaining for children who rely more heavily on the caregiver’s assistance. Conversely, the use of PlayCuff may have required a steeper learning curve for optimal use, not achievable within a single gaming session. The higher usability reported for PlayCuff by children with lower levels of independence is likely due to the fact that controlling the game with PlayCuff does not require grasping or handling any manipulandum. Additionally, the orthosis stabilizes posture, thereby aiding in reducing errors.

Notably, our study found that perceived usability and acceptability of GiocAbile were consistently good across children with different levels of impairment. This suggests that GiocAbile has the potential to be widely applicable and enjoyable for children with CP, irrespective of GMFCS, MACS, and ICF scores, underscoring its versatility and potential as a universally applicable tool in pediatric motor rehabilitation.

As recently emphasized by the Director of Accessibility of Xbox at Microsoft [32], the presence of barriers to play, such as a lack of accessibility or inclusivity in a game, can deter users from playing with it. Consequently, even if a serious game has excellent rehabilitation potential, its effectiveness may be limited by its ability to engage the child.

In our study, correlations were observed between specific impairment measures and user experience dimensions. For instance, greater cognitive impairment (i.e., higher cognitive ICF) was associated with higher levels of satisfaction during gameplay, indicating that more severely cognitively impaired children may derive greater satisfaction from the gaming experience. Since individuals with cognitive disabilities may encounter obstacles that influence their ability to understand and engage in video games, it is probable that their satisfaction is compromised when games fail to accommodate their special needs [33]. Conversely, when video games are designed with consideration for the unique needs and challenges of individuals with disabilities, like in GiocAbile’s case, it is likely that their satisfaction and overall gaming experience will be enhanced.

Finally, in our study we found that children with higher MACS scores, indicating more severe manual impairments, found GiocAbile easier to use compared to children with lower MACS scores. This may be due to the low expectations that children with severe manual impairment may have towards playing video games. Consequently, being able to play and engage with GiocAbile may have made them perceive the system as easy to use (i.e., easier than expected). On the other hand, unsurprisingly, children with higher MACS scores felt less control over the game than children with less severe manual impairment. Nevertheless, the *Self Efficacy* construct of the TAM3 highlighted a general perceived lack of capacity to use the system. However, the high level of engagement observed among all participants, regardless of their MACS score, supports our hypothesis that with more gaming sessions even children with greater manual impairments may improve their proficiency with GiocAbile.

Based on the existing literature [6, 7, 34, 35], we can speculate that, by providing a user-friendly and enjoyable platform for engaging in therapeutic activities, GiocAbile has the potential to enhance motivation, participation, and ultimately outcomes in rehabilitation programs, although further studies are needed to confirm our hypothesis. Furthermore, from a practical standpoint, our findings imply that GiocAbile could be seamlessly integrated into existing rehabilitation protocols without the need for extensive customization or adaptation based on individual level of impairment. This could streamline the implementation process and reduce barriers to adoption, making it more feasible for healthcare providers to incorporate GiocAbile into their practice.

### Future perspectives

The evaluation of the rehabilitative effectiveness of GiocAbile was beyond the scope of the present pilot study but will be the subject of future investigations. Furthermore, future research will focus on exploring the feasibility and user experience of the multiplayer mode, which could enhance social integration among children with CP, allowing them to engage in gaming experiences with peers, regardless of their level of impairment. Finally, previous studies have evaluated the use of affordable motion interactive games as a home-based intervention, underscoring the practicality of integrating gaming technology into the daily routines of children with neuromotor disabilities [8]. Future studies may explore the feasibility of GiocAbile in a home setting, as well.

### Study limitations

To ensure a proper interpretation of our results, some limitations need to be acknowledged. Firstly, our study’s small sample size, while consistent with similar research in the field [6], may hinder the generalizability of our results. Additionally, the use of a single gaming session, though common in comparable studies [6], further underscores the need for caution in extrapolating our findings. Indeed, engaging with GiocAbile for the first time and for a short period of time may have heightened the excitement of children due to the novelty of the experience. Further studies with more and longer gaming sessions are needed to confirm our preliminary results.

Furthermore, our study population was limited to children with CP falling within specific ranges of GMFCS, MACS, and ICF scores. This may limit the applicability of our findings to children with other diagnoses or with more severe levels of impairment. Finally, it is important to note that the ad hoc questionnaire utilized in our study has not undergone validation. Consequently, it may lack proven accuracy and reliability in assessing respondents’ evaluations of GiocAbile’s software and hardware features. Moreover, despite efforts such as language revisions and the inclusion of emoticons to enhance clarity considering the age of the study population, a residual risk of misinterpretation or misunderstanding of questions cannot be excluded.

## Conclusions

The evaluation of acceptability, usability, and user experience of the video game GiocAbile among children with CP yielded promising results. Despite the varying degrees of disability among participants, GiocAbile was found to be highly accessible, usable and enjoyable. This suggests that GiocAbile has the potential to be effectively utilized by children with diverse levels of impairment, highlighting its versatility and adaptability. Further research is needed to explore its rehabilitative potential and to address the specific needs of different user populations.

## Electronic Supplementary Material

Below is the link to the electronic supplementary material.

Supplementary Material 1

Supplementary Material 2

Supplementary Material 3

Supplementary Material 4

Supplementary Material 5

## Data Availability

The datasets supporting the conclusions of this article are included within the article (and its additional files).

## Abbreviations

APS: Assistance to Participate Scale
CP: Cerebral Palsy
GMFCS: Gross Motor Function Classification System
ICF: International Classification of Functioning, Disability, and Health
MACS: Manual Ability Classification System
SUS: System Usability Scale
TAM3: Technology Acceptance Model 3
