# Supplementary material for: Inclusivity is child’s play: pilot study on usability, acceptability and user experience of a sensory-motor PC game for children with cerebral palsy (GiocAbile)
Source: Ital J Pediatr. 2024 Dec 20;50:263. doi: 10.1186/s13052-024-01830-7 (PMC11662457; doi:10.1186/s13052-024-01830-7)
Supplement: Supplementary file 1 — Supplementary Material 1 [file 13052_2024_1830_MOESM1_ESM.docx]

***Supplementary Table 2:*** *Baseline clinical evaluations and APS total score of the study population* *(Subjects 01-19).*

| **ID** | **GMFCS** | **MACS** | **Cognitive ICF** | **Motor ICF** | **APS** |
| --- | --- | --- | --- | --- | --- |
| **S01** | 5 | 3 | 2 | 3 | 66 |
| **S02** | 4 | 3 | 2 | 3 | 72 |
| **S03** | 2 | 1 | 2 | 1 | 67 |
| **S04** | 1 | 1 | 1 | 0 | 67 |
| **S05** | 2 | 1 | 3 | 2 | 60 |
| **S06** | 1 | . | 2 | . | 64 |
| **S07** | 1 | 1 | 1 | 0 | 75 |
| **S08** | 4 | 3 | 1 | 3 | 54 |
| **S09** | 3 | 2 | 3 | 3 | 60 |
| **S10** | 1 | 2 | 3 | 1 | 72 |
| **S11** | 2 | 1 | 1 | 2 | 80 |
| **S12** | 2 | 2 | 2 | 2 | 66 |
| **S13** | 4 | 3 | 2 | 3 | 60 |
| **S14** | 2 | 2 | 0 | 2 | 72 |
| **S15** | 1 | 3 | 1 | 1 | 57 |
| **S16** | 1 | 3 | 1 | 1 | 66 |
| **S17** | 1 | 3 | 1 | 1 | . |
| **S18** | 1 | 2 | 0 | 0 | 67 |
| **S19** | 1 | 2 | 0 | 0 | 88 |
