# Supplementary material for: Inclusivity is child’s play: pilot study on usability, acceptability and user experience of a sensory-motor PC game for children with cerebral palsy (GiocAbile)
Source: Ital J Pediatr. 2024 Dec 20;50:263. doi: 10.1186/s13052-024-01830-7 (PMC11662457; doi:10.1186/s13052-024-01830-7)
Supplement: Supplementary file 2 — Supplementary Material 2 [file 13052_2024_1830_MOESM2_ESM.docx]

***Supplementary Table 1:*** *Children’s responses to the ad hoc questionnaire.*

| **QUESTIONS** | **ANSWERS** | | | | | |
| --- | --- | --- | --- | --- | --- | --- |
|  | **1** | **2** | **3** | **4** | **5** | **NA** |
| I found the game storyline interesting | 0 | 0 | 0 | 5 | 6 | 8 |
| I identified with Mako the monkey | 7 | 1 | 1 | 5 | 4 | 1 |
| I liked Mako the monkey | 1 | 1 | 1 | 4 | 9 | 3 |
| I liked the platform-game environments:  - Jungle  - Water  - Desert  - Sky  - Lands of ice  - Mountains | 1  2  1  1  0  0 | 0  1  0  1  0  0 | 0  1  0  0  0  0 | 4  2  0  0  1  2 | 13  12  4  2  1  1 | 1  1  14  15  17  16 |
| I liked the minigames:   - Canoeing - Boat - Drill - Flight - Fishing - Climbing - Crystal assembly | 0  1  0  4  1  1  2 | 1  1  0  0  0  0  1 | 2  1  1  1  0  0  1 | 5  4  5  3  6  6  6 | 9  6  12  9  6  3  3 | 2  6  1  2  6  9  6 |
| I liked the sounds played during gameplay | 2 | 0 | 2 | 6 | 7 | 2 |
| The game explained clearly how to use the controllers | 0 | 0 | 1 | 1 | 7 | 10 |
| I managed to use the controllers:   - PlayCuff - Double lever - Double crank - Single crank and lever - Steering wheel | 0  0  0  0  1 | 1  1  0  1  0 | 1  0  0  0  1 | 8  5  3  3  3 | 9  10  6  11  13 | 0  3  10  4  1 |
| I managed to get the controllers to do what I wanted:   - PlayCuff - Double lever - Double crank - Single crank and lever - Steering wheel | 1  0  0  0  0 | 1  1  0  0  0 | 1  1  0  1  1 | 7  5  5  6  4 | 5  6  3  6  9 | 4  6  11  6  5 |
| Using the controllers was fun:   - PlayCuff - Double lever - Double crank - Single crank and lever - Steering wheel | 1  1  0  0  1 | 3  0  0  0  0 | 0  0  0  0  1 | 2  2  2  3  2 | 12  13  5  11  13 | 1  3  12  5  2 |
| Using the controllers was disappointing:   - PlayCuff - Double lever - Double crank - Single crank and lever - Steering wheel | 9  11  4  9  12 | 1  0  1  1  1 | 0  1  0  0  0 | 1  0  0  0  0 | 1  1  1  0  0 | 7  6  13  9  6 |
| Using the controllers was complex:   - PlayCuff - Double lever - Double crank - Single crank and lever - Steering wheel | 9  9  3  7  12 | 1  0  0  0  0 | 0  1  2  1  1 | 5  1  1  2  1 | 1  3  0  2  0 | 3  5  13  7  5 |
| PlayCuff was uncomfortable to wear | 12 | 1 | 0 | 3 | 2 | 1 |
| PlayCuff helped me finish the game/make less mistakes | 2 | 0 | 1 | 4 | 11 | 1 |
| I felt frustration/disappointment while playing | 10 | 1 | 0 | 0 | 0 | 8 |
| I felt satisfied while playing | 0 | 0 | 0 | 4 | 10 | 5 |
| I would recommend this video game to a friend | 3 | 0 | 0 | 5 | 10 | 1 |
