# Supplementary figures and images for: Inclusivity is child’s play: pilot study on usability, acceptability and user experience of a sensory-motor PC game for children with cerebral palsy (GiocAbile)
Source: Ital J Pediatr. 2024 Dec 20;50:263. doi: 10.1186/s13052-024-01830-7 (PMC11662457; doi:10.1186/s13052-024-01830-7)

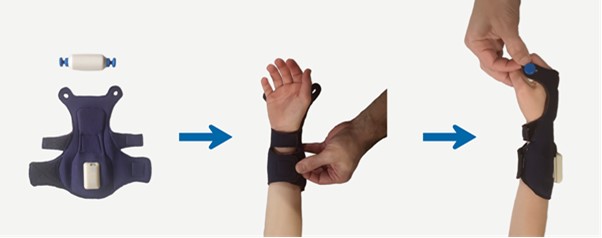

Supplement: Supplementary file 3 — Supplementary Material 3 [file 13052_2024_1830_MOESM3_ESM.jpg]

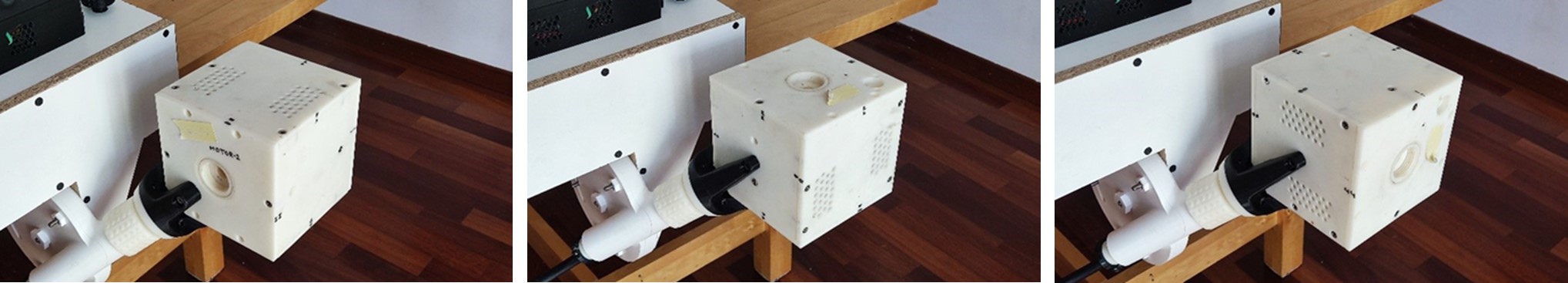

Supplement: Supplementary file 4 — Supplementary Material 4 [file 13052_2024_1830_MOESM4_ESM.jpg]

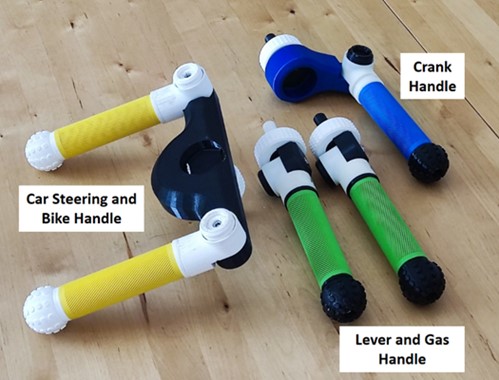

Supplement: Supplementary file 5 — Supplementary Material 5 [file 13052_2024_1830_MOESM5_ESM.jpg]
